# Emerging Evidence of Epigenetic Modifications in Vascular Complication of Diabetes

**DOI:** 10.3389/fendo.2017.00237

**Published:** 2017-09-29

**Authors:** Madhu Khullar, Balneek Singh Cheema, Satish K. Raut

**Affiliations:** ^1^Department of Experimental Medicine and Biotechnology, Postgraduate Institute of Medical Education and Research, Chandigarh, India; ^2^PAREXEL International, Chandigarh, India

**Keywords:** diabetes, cardiovascular complication, epigenetics, DNA methylation, histone modifications, non-coding RNAs

## Abstract

Genes, dietary, and lifestyle factors have been shown to be important in the pathophysiology of diabetes and associated microvascular complications. Epigenetic modifications, such as DNA methylation, histone acetylation, and post-transcriptional RNA regulation, are being increasingly recognized as important mediators of the complex interplay between genes and the environment. Recent studies suggest that diabetes-induced dysregulation of epigenetic mechanisms resulting in altered gene expression in target cells can lead to diabetes-associated complications, such as diabetic cardiomyopathy, diabetic nephropathy, retinopathy, and so on, which are the major contributors to diabetes-associated morbidity and mortality. Thus, knowledge of dysregulated epigenetic pathways involved in diabetes can provide much needed new drug targets for these diseases. In this review, we constructed our search strategy to highlight the role of DNA methylation, modifications of histones and role of non-coding RNAs (microRNAs and long non-coding RNAs) in vascular complications of diabetes, including cardiomyopathy, nephropathy, and retinopathy.

## Introduction

In spite of adequate glycemic control, incidence of vascular complications associated with diabetes, such as diabetic cardiomyopathy, retinopathy, nephropathy, and neuropathy, remains high contributing to increased morbidity and mortality in diabetic patients. Recent studies suggest that a complex interplay between genes and environment may significantly contribute to pathogenesis of microvascular complications associated with diabetes (1–3). Emerging evidence suggests that environmental factors modulate aberrant expression of several key genes through epigenetic mechanisms in type II diabetes mellitus (T2DM) (4). Epigenetic changes, such as DNA methylation, histone modifications, and interference of RNAs, comprise the major epigenetic regulators of gene expression. A large volume of data has emerged supporting aberrant DNA methylation, histone modifications, and expression of microRNAs and long non-coding RNAs (lncRNAs) contributing to deregulation of signaling pathways (oxidative stress, inflammation, and apoptosis, etc.) in T2DM. However, our knowledge on epigenetic regulation in diabetes-associated microvascular complications remains limited. Thus, elucidation of epigenetic changes could provide better understanding of pathophysiology and therapeutic management of these diseases. In this article, we briefly summarize recent findings on the role of DNA methylation, histone modifications, and post-transcriptional RNA regulation in microvascular complications of diabetes.

## Search Methodology

Literature searches of several electronic databases including Embase, Google Scholar, Ovid SP, Pubmed/Medline, and Web of Science were searched using the following search terms (free text, truncation, and MeSH or EMTREE terms): “DNA methylation” OR “histone acetylation,” non-coding RNAs, microRNAs, long non-coding RNAs, post-transcriptional RNA regulation, epigenetic modifications, vascular, cardiovascular, renal, and retinal complications of diabetes for relevant publications in English language from 2006 to till date to evaluate the association between the role of DNA methylation, post-transcriptional RNA regulation, and histones modifications in diabetes-associated microvascular complications. Reference lists of included studies were hand-searched to identify other potentially eligible studies. Three authors (Madhu Khullar, Satish K. Raut, and Balneek Singh Cheema) reviewed the titles and abstracts to identify potentially eligible papers. These papers were examined in full detail. Final decision regarding inclusion was resolved by discussion. A manual review has been used for related publications and references of retrieved articles. We included randomized or non-randomized controlled clinical trials with or without blinding as well as cross-sectional and interventional studies that provided sufficient information.

## Epigenetic Modifications in Diabetes-Induced Microvascular Complications

**Figure 1:**
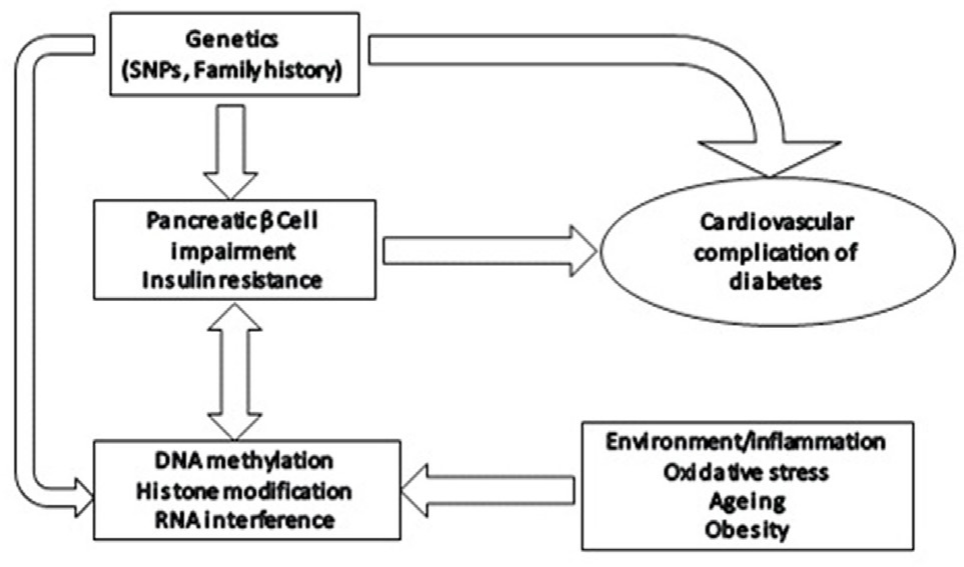
Epigenetic modifications in diabetes: effect of various environmental/physiological factors on gene expression through epigenetic modifications, such as altered DNA methylation, histone modifications, and post-transcriptional RNA regulation.

Evidence from both animal studies and clinical studies in diabetic patients has provided strong evidence linking histone modifications, post-transcriptional RNA regulation, and DNA methylation in microvascular complications of diabetes by regulating molecular pathways involved in pathophysiology of microvascular complications in diabetes (Figure 1).

These changes are inheritable and persist even after adequate glycemic control and contribute to metabolic memory and have been suggested to significantly contribute to diabetes-induced vascular complications (5).

## Histone Modifications in Diabetes-Induced Microvascular Complications

Histone acetyl transferases (HATs) and histone deacetylases (HDACs) are the enzymes involved in histone acetylation/deacetylation and have been recently shown to be involved in regulating gene expression of several key molecules involved in microvascular complication of diabetes (6).

## HDACs in Diabetes-Induced Microvascular Complications

Histone deacetylases silence gene expression by deacetylating histone tails resulting in condensed euchromatin. Recent studies have implicated HDACs in diabetes and its associated microvascular complications; for example, HDAC1 and HDAC2 were shown to modulate expression of cardiac hypertrophy genes (6).

O-linked β-N-acetylglucosamine (O-GlcNAc) is an important signaling molecule which regulates cell function through O-GlcNAcylation of serine and threonine residues of proteins (7). O-GlcNAc plays a central role in regulating cardiovascular function. Increased O-GlcNAc levels observed in diabetic hearts and have been linked to diabetic cardiomyopathy (8). Cox and Marsh have reported decreased levels of Mammalian switch-independent 3 A (*mSin3A*), *HDAC1, HDAC2*, and increased expression of *HDAC2* mRNA and HDAC1/2 deacetylase activity in hearts from diabetic rats. These authors have suggested that there is a decreased physical association of O-GlcNAc with mSin3A/HDAC1/2 in the heart which results in their altered activity and expression in the diabetic heart and impacts its function. However, physical exercise increased cardiac O-GlcNAc of these proteins resulting in beneficial effects on cardiac function and proposed that anti-hypertrophic effects of exercise on diabetic hearts were mediated by O-GlcNAc mediated post translation modification of HDAC1, 2 and mSin3A (9).

HDAC3 has been shown to exert pro-hypertrophic effect in diabetic mice. Xu et al. have reported significantly increased cardiac HDAC3 activity in the OVE26 diabetic mice. They showed that HDAC3 was exerting its pro-hypertrophic activity by downregulating DUSP5 (a MAP Kinase phosphatase) expression, by deacetylation of histone H3 in the primer region of *DUSP5* gene (10). There are several studies showing beneficial and preventive effects of HDAC inhibition on diabetes-induced cardiovascular function. (This has been given in detail in a separate section.) However, further research is warranted to identify the specific HDAC isoforms that are dysregulated and their molecular targets that result in diabetic cardiomyopathy.

The role of HDACs in diabetic nephropathy has been reviewed recently by Li et al. (11). The available literature suggests that different HDAC isoforms targeting different molecular pathways are involved in pathophysiology of diabetic nephropathy. For example, HDAC1, HDAC2, and HDAC5 were shown to modulate expression of genes induced by TGF-β1 (12), TGF- β (13), and HDAC4 inhibited autophagy by deacetylating STAT1 (14).

Increased histone acetylation has been also reported in diabetic retinopathy and has been partly attributed to high glucose-mediated decreased HDAC activity in retinal cells. HDAC activators and HDAC inhibitors were found to mitigate or potentiate diabetes-induced histone acetylation and expression of pro-inflammatory proteins in high glucose-treated cultured retinal Müller glia cells, confirming contribution of histone acetylation of retinal cells in pathophysiology of diabetic retinopathy (14). Decreased IL-10 levels are seen in diabetic retinopathy patients that have been suggested to be due to increased HDAC11 activity in conjunction with miR-19a in peripheral B cells of diabetic retinopathy patients (15).

Thus, available evidence supports a pathogenic role for aberrant HDAC activity in DC, DN, and DR by promoting histone acetylation and repression of genes of various signaling pathways, such as pro-inflammatory, pro-fibrotic, and antioxidant pathways.

## HATs in Diabetes-Induced Microvascular Complications

Histone acetylation mediated by HATs is another important epigenetic mechanism in gene regulation. HATs acetylate specific lysine residues of core histones at the N-terminal tail, causing DNA uncoiling, increased accessibility to transcription factors, and increased gene expression. Thus, altered HAT activity could regulate gene expression and affect cell function. Indeed, HATs have been implicated in several diseases, such as cancer, diabetes, cardiac hypertrophy, asthma, and so on.

*Recent evidence suggests that HATs may participate in the pathophysiology of microvascular complications of diabetes by regulating the expression of inflammatory pathway genes*. For example, high glucose treatment of monocytes was found to increase transcriptional activity of HATs CBP and p/CAF, resulting in increased histone lysine acetylation of promoter regions of inflammatory genes, cyclooxygenase-2 (*COX-2*) and *TNF-*α gene, and increased gene expression of these cytokines in cultured monocytes (16). An increased promoter histone lysine acetylation of inflammatory genes has been reported in monocytes from both T1DM and T2DM patients (17). Furthermore, HAT-mediated lipid oxidation has been also found to increase inflammation by increasing histone acetylation of inflammatory genes (18). Yun et al. observed that HATs-mediated increased pro-inflammatory cytokine expression could be attenuated by curcumin in high glucose-treated human monocytes (19). *Curcumin was shown to decrease high glucose-induced HAT activity, p300 gene expression, and acetylation of CBP/p300, a complex that functions as a coactivator of NF-κB*. The role of HATs in diabetic nephropathy has been recently reviewed by Li et al. (11) and provides evidence that high glucose-induced increased activity and levels of HATs, such as p300, CBP, and p/CAF, are mediating the activation of pro-inflammatory cytokines, ECM proteins, endothelial function, and fibrotic processes in diabetic nephropathy, *via* acetylation of both histone and non-histone proteins, such as Smads, p53, SP1, and NF-κB.

## Sirtuins in Diabetes-Induced Microvascular Complications

Recently, another class of HDACs, Sirtuins has been shown to regulate key cellular and metabolic processes by deacetylating the lysine residues of proteins involved in these processes. Sirtuins are a highly conserved protein family of HDACs and have been found to have protective effects against several diseases, such as diabetes, cancer, cardiovascular, and neurodegenerative diseases (20). Sirtuins exert these beneficial effects by modulating the expression of the genes involved in energy metabolism, DNA repair, inflammation, fibrosis, and oxidative stress (21).

Sirtuins regulate enzymes of carbohydrate metabolism, lipid metabolism, adipogenesis, and insulin secretion in diabetic patients (15). SIRT1 regulates glucose metabolism in liver, pancreas, muscle, and adipose tissue, mainly by regulating PGC-1α (22). SIRT1 induces gluconeogenic genes through deacetylation of PGC-1α in fasting state. FOXO group of transcriptional factors promote gluconeogenesis *via* STAT3; SIRT1 inhibits gluconeogenesis by inhibiting gluconeogenic genes *via* deacetylation of FOXO transcription factors and STAT3 in liver (22). Increased SIRT1 is also shown to increase glucose-induced insulin secretion in pancreatic β-cells which is partly due to SIRT1-mediated inhibition of UCP-2 in pancreatic islet β-cells (22). SIRT3, a mitochondrial protein deacetylase was found to be effective in increasing insulin sensitivity and decreasing serum glucose (16). SIRT4, another sirtuin involved in glucose homeostasis acts by repressing enzyme glutamate dehydrogenase (GDH) inhibiting insulin secretion (23). Decreased levels of SIRT1, 3, and 4 have been observed in diabetic patients and were associated with hepatosteatosis. Apart from this, sirtuins have been also shown to regulate activity of NFkB and expression of its downstream inflammatory genes in diabetes (18, 19).

Recent studies show that cardiac Sirtuins expression is dysregulated in diabetic patients. Bagul et al. reported a decrease in cardiac SIRT-1 and increase in SIRT-3 activity in the T2DM rat and downregulation of all sirtuins except SIRT-2, which was increased in T1DM rat heart (24). In a recent review on sirtuins in cardiac complications of diabetes, sirtuins were suggested to attenuate the effects of insulin resistance and oxidative stress pathways in heart (25). SIRT-1 has been found to be the most important modulator of vascular function and is being targeted for therapeutic potential in various pre-clinical studies to improve cardiovascular functions. Bagul et al. have recently shown beneficial effect of reservatol on diabetic rat heart through modulating expression of SIRT-1 in T2DM and SIRT-1, 2, 3, and 5 in T2DM (24).

Recently, role of Sirtuins in vascular homeostasis has been reviewed nicely (26). Sirtuins were shown to regulate endothelial damage and vascular repair mechanisms. Sirtuins, by acting on specific endothelial targets, regulate several processes, including inflammation by modulating cytokine expression (IL-6, TNF-α, NF-KB, MMP-14), oxidative stress [manganese superoxide dismutase (MnSOD), FOXOs], and deacetylation of histone H3K14 and H4K16 (27).

High glucose milieu has been found to induce endothelial cell senescence and functional abnormalities by repressing SIRT1 expression high glucose-treated endothelial cells. SIRT1 upregulation in these cells was found to be protective against glucose-induced endothelial dysfunction indicating its potential protective role in diabetic vascular complications (21). Advanced glycation end products (AGEs), important mediators of diabetes, induced vascular abnormalities. AGEs have been shown to decrease SIRT1 levels and promote apoptosis in human endothelial Eahy926 cells which could be reversed by increasing SIRT1, confirming that AGEs were inducing apoptosis by repressing SIRT1 in endothelial cells (28). The downregulation of SIRT1 by high glucose and in diabetic has been proposed to be mediated by glucose-induced oxidative stress in endothelial cells. Mortuza et al. showed that high glucose-induced downregulation of SIRT1 was accompanied by FOXO1-mediated decreased levels of antioxidant enzyme, suggesting that SIRT1/FOXO1 axis was regulating oxidative status in endothelial cells (27).

Decreased SIRT1 expression has been also implicated in increased cellular senescence in renal glomerulus and retinal blood vessels in diabetic male C57BL/6 mice which were mediated by p300 and FOXO1 mediated reduction in mitochondrial antioxidant enzyme MnSOD in these cells (27). Furthermore, SIRT1 overexpression has been also shown to be protective in diabetes-induced renal and retinal injury in diabetic mice, through attenuated p300, endothelin-1 (ET-1), and TGF-β1 expression (29). Downregulation of SIRT1 has been also shown to promote diabetic retinopathy by inducing increased MMP-9 expression in retinal endothelial cells (RECs) *via* acetylating transcriptional factor AP-1 (30). AGEs have been recently reported to decrease SIRT3 levels and SIRT3 knock down was associated with endothelial dysfunction in endothelial progenitor cells (EPCs). Moreover, SIRT3 augmentation ameliorated cellular dysfunction and enhanced antioxidant machinery (31). SIRT6 deficiency has been found to impair wound healing in diabetic db/db mice and induce pro-inflammatory cytokines and oxidative stress, and decrease angiogenesis, suggesting its potential role in diabetic vasculopathy (32).

The role of other sirtuins in diabetic vascular complications is not known and needs to be investigated. Overall, diabetes-induced downregulation of sirtuins (SIRT1, 3 and 6) appears to promote oxidative stress and endothelial dysfunction, and induce cellular fibrosis, suggesting these molecules to be of potential therapeutic use in diabetes and associated vascular complications.

## Histone Methylation in Diabetes-Induced Microvascular Complications

Methylation of core histone tails at lysine or arginine residues are known to modulate gene expression by changing chromatin structure. For example, methylation at H3-K9 and H3-K27 mediates heterochromatin formation and results in silencing gene expression. Aberrant histone lysine methylation has been found to be involved in several pathological processes such as cancer, diabetes, cardiovascular diseases, etc. High glucose has been shown to induce increased histone H3 lysine 9 dimethylation in THP1 monocytes. Miao et al. showed that high glucose exposure caused increased H3K4me2 and H3K9me2 of specific chromatin regions and their associated genes. They reported increased H3K4me2 was associated with increased methylation of nine genes, including *ICAM3, FOS, GSTA-4, IL-8*, and *BCL-9*, showed decreased methylation following HG exposure. Similarly, H3K9me2 methylation resulted in increased methylation of 39 genes and decreased methylation of 11 genes. They further observed increased H3K9me2 at the coding and promoter regions of two candidate genes (*IL-1A* and *PTEN*) in blood monocytes of diabetic patients, indicating that diabetic milieu induced aberrant histone methylation is an important contributor to diabetes-associated complications (33).

Histone methyl transferases (HMTs) carry out methylation at specific lysine or arginine residues. HMTs Suv39 and G9a family methylate histone H3 at Lys9 and cause gene silencing whereas SET1/2 family HMTs methylate histone H3 at Lys4 and correlate with gene activation. Okabe et al. reported sustained vascular gene expression of H3K4 methyl transferase, Set7 as a responsive measure to hyperglycemia in vascular endothelial cells. They showed that metabolic memory of prior exposure to hyperglycemia was induced by Set7 and proposed that Set7 was a potential molecule for the phenomenon of hyperglycemic memory (34). This was further supported by an another study which showed that high glucose exposure altered ratio of cytoplasmic/nuclear ratio of Set7 protein without changing overall level of Set7 in vascular endothelial cells, indicating a role of Set7 and its role in hyperglycemia-induced gene activation of vascular endothelial cells (35).

The role of histone methylation in diabetic retinopathy has been also documented. For example, Zhong et al. showed that retinal superoxide dismutase gene (*SOD2*) was epigenetically regulated in diabetes through methylation/acetylation of H4K20me3, acetyl H3K9, and NF-kB p65 on the histones at the promoter/enhancer location of retinal *SOD2* in diabetes (36). These authors showed that these modifications continued after termination of hyperglycemia, supporting a diabetes-induced epigenetic regulation of retinal *SOD2* (36). Their study suggests that promoter region methylation of *SOD2* histones might play an important role in progression of diabetic retinopathy. Similarly, H3K9-specific demethylase JHDM2A (also known as JHMJD1A and KDM3A) has also been shown to be involved in regulating the expression of metabolic genes, strengthening the role of epigenetic regulation of metabolic genes in microvascular complications of diabetes (37). These authors observed that JHDM2A regulates the expression of PPARα and β-adrenergic signaling pathway genes and suggested that JHDM2A might regulate energy mediated β-adrenergic signaling pathway (37).

The fetal exposure to maternal milieu such as nutrition is known to result in intrauterine growth restriction (IUGR) and influence susceptibility to several diseases such as insulin resistance in adults. Hepatic insulin growth factor 1 (IGF-1) modulates insulin sensitivity, thus decreased IGF-1 levels are linked to insulin resistance. Decreased post natal plasma IGF-1 levels have been reported in IUGR infants and in new born rats with induced IGUR (38). Fu et al. have shown that IUGR affects *IGF-1* gene expression by modulating the region and gender-specific histone modifications (methylation and acetylation) along the length of *IGF-1* gene. The authors showed that IUGR significantly increased H3K4me2 in males and H3 K4me3 in females new born rats with induced IUGR. Since, there is a dynamic association between histone methylation and associated DNA methylation which affects gene transcription, these histone modifications resulted in decreased IGF-1 expression in new born rats. These findings suggest that that aberrant methylation of core histone tails of hepatic IGF-1 regulate IGF-1 expression.

Yu et al. (39) have shown that combination of diabetes and renal failure accelerated cardiomyopathy by epigenetic alterations (increased acetylation, phosphorylation, K4 dimethylation, and reduced K9 dimethylation) of the cardiac histones H3. They observed increased H3 dimethylation at lysine 4 and 9 and decreased H3 dimethylation at lysine 9 in hearts of uni-nephrectomized db/db mice resulting in transcriptionally active chromatin and proposed that these changes were associated with increased expression of cardiac hypertrophy related genes. However, factors causing these changes are not known and need to be determined.

Histone methylation in etiology of diabetic nephropathy has been widely investigated and reviewed recently (40). Diabetic nephropathy is characterized by glomerular mesangial expansion, inflammation, renal fibrosis, and hypertrophy. In a recent study, Li et al. (41) showed that increased p21 expression seen in high glucose-treated mesangial cells was mediated by reduced histone H3-lysine9-dimethylation (H3K9me2), increased histone H3-lysine4 methylation (H3K4me1/3) and increased translocation of SET7/9 at the p21 promoter region. Similarly, Yuan et al. (12) also showed that oxidized lipid products such as 2(S)-hydroxyeicosatetraenoic acid [12(S)-HETE] increased transcriptional activity of SET7, which in turn increased expression of pro-fibrotic genes in HETE treated mesangial cells. Losartan, an AT1R inhibitor, a common drug used in treatment of diabetic nephropathy has been shown to decrease H3K9/14Ac at RAGE, PAI-1, and MCP-1 promoters, in mesangial cells from db/db diabetic mice, suggesting that AT1R action may be also mediated by attenuation of epigenetic changes of the key genes involved in diabetic nephropathy.

Altered histone methylation of RECs has been reported in diabetic retinopathy. For example, decreased expression of MnSOD was found to be associated with altered H3K4me1/me2in diabetic retinas and endothelial cells (42). These changes were found to persist even after normalization of blood glucose levels, indicating that these changes acted as markers of metabolic memory (42).

Decreased H3K9me2 promoter methylation of MMP9, promoting increased expression has been also seen in diabetic retinas and suggested to be associated with increased ECM accumulation (42). Increased expression of PRMT4, a methyltransferase which specifically methylates H3R17 histones and promotes cell death has been observed in retinal pigment epithelial layer of diabetic rats even before development of diabetic retinopathy (43). Wang et al. (44) reported differential methylation on H3, H4, H2A, H2B, and H1 sites in diabetic retinas specifically they observed increased mono- and dimethylation of histone H4 lysine 20 (H4K20me1/me2), and were associated with DNA damage in retinas of diabetic rats and these methylation patterns could be partly reversed by minocycline, a strong neuroprotective drug and used in treatment of diabetic retinopathy. Thus, altered histone methylation appears to be important in development of diabetic retinopathy in animal models and *in vitro* conditions, however, these changes need to be replicated in diabetic patients.

Thus, in summary, hyperglycemia-induced differential histone methylation/acetylation appears to regulate expression of several genes of cellular pathways, such as endothelial activation, oxidative stress, adrenergic signaling pathway, and so on, involved in diabetes-induced vascular complications.

## Modulation of HDACs and HATs as a Therapeutic Approach

Since HDACs along with HATs have been shown to have a critical role in regulating expression of genes involved in diabetic vascular complications, modulation of these molecules is being investigated for therapeutic applications in diabetic cardiomyopathy, nephropathy, retinopathy, and endothelial dysfunction associated with diabetes (45).

For example, acetylation of 20 S proteasome subunits in the heart has been shown to mediate proteolytic activity of injured myocardium (46), it has been suggested that modulation of HDACs, the key regulators of acetylation in the cell could be used effectively in the treatment of cardiac injury (47, 48). Christensen et al. reported that HDAC inhibition could ameliorate late diabetic microvascular complications along with improving insulin resistance and β-cell function (49). HDAC inhibition was also shown to improve cardiac function and attenuated cardiac remodeling in the diabetic myocardium of the streptozotocin-treated ICR mice. Chen et al. observed that diabetic mice given 1% butyrate in drinking water resulted in HDAC inhibition in the diabetic myocardium, specifically myocardial HDAC4 was found to be significantly decreased. HDAC inhibition caused upregulation of GLUT 1 and 4, increased Caspase 3, increased myocardial superoxide dismutase, decreased cardiac interstitial fibrosis and myocyte hypertrohy resulting in improvement in cardiac performance in diabetic mice (50).

Chen et al. have also recently shown that HDAC inhibition promotes stem cell-derived myocardial repair, thereby improving cardiac function and attenuating cardiac remodeling in diabetic rats, further confirming a protective role of HDAC inhibitors against myocardial injury (50).

Peroxisome proliferator-activated receptors (PPARs) play an important role in diabetes-associated heart diseases by regulating cardiac glucose and lipid homeostasis. HDAC inhibitor, MPT0E014, was shown to decrease cardiac inflammation and dyslipidemia by modulating myocardial PPARs, and attenuated diabetic cardiomyopathy (51).

DUSP 5 is a dual-specific phosphatase which dephosphorylates and inactivates ERK1/2 MAP Kinase, a known promoter of cardiac hypertrophy (10). Xu et al. recently reported that HDAC3 inhibition with its selective inhibitor, RGFP966, increased the expression of MAP kinase phosphatase, DUSP 5 and prevented development of diabetic cardiomyopathy in Type 1 diabetes OVE26 mice, suggesting a therapeutic potential of HDAC3 inhibition in prevention of diabetic cardiomyopathy (10).

Histone deacetylase inhibitors have been also found to be effective in preventing diabetes-induced renal damage. Gilbert et al. (52) reported that HDAC inhibitor, Vorinostat, blunted renal damage in diabetic rats by reducing renal growth and glomerular hypertrophy *via* modulating renal EGFR expression. Vorinostat has been also shown to attenuate renal damage in strptozotocin-treated mice by decreasing eNOS expression and oxidative stress (53). Valproic acid (VPA), a known HDAC inhibitor also has been shown to ameliorate diabetes-induced renal injury by inhibiting renal fibrosis (54). Increased oxidative stress is an important contributor to diabetic nephropathy; Dong et al. recently showed that sodium butyrate inhibited HDAC activity and elevated the expression of *NRF2* and its downstream targets heme oxygenase 1 and NAD(P)H dehydrogenase quinone 1. Deletion of the *NRF2* gene completely abolished sodium butyrate activation of NRF2 signaling and protection against diabetes-induced renal injury (55). Trichostatin A (TSA), an antifungal antibiotic has been shown to inhibit HDACs 1, 3, and 4. TSA suppresses redox signaling by decreasing NADPH Oxidase 4 (Nox4) expression by inhibiting p300-HAT-dependent pathway in endothelial cells (56). Cao et al. showed that TSA decreased transverse aortic constriction (TAC), induced cardiac hypertrophy and phenylephrine (PE) or ET-1, and induced cardiomyocyte hypertrophy by inhibiting autophagy, and suggested that TSA-mediated HDAC inhibition suppresses load- or agonist-induced autophagy in stressed myocardium (57).

Pancreatic duodenal homeobox 1 (PDX1) is a transcription factor associated with pancreatic β-cell function and survival. PDX1 deficiency results in defective B-cell function and diabetes. Park et al. observed that IUGR decreased fetal and postnatal PDX1 levels by histone modification of *PDX1* gene in primary islets. IUGR promoted deacetylation of histones H3 and H4 by recruiting HDAC1 and corepressor Sin3A; and histone 3 lysine 4 (H3K4) was demethylated and histone 3 lysine 9 (H3K9) was methylated, resulting in silencing of the PDX1. These authors suggested that IUGR-induced *PDX1* gene silencing in the β cell was linked with development of T2DM (58).

Johnson and Marsh recently reported that treatment of Type 2 diabetic db/db mice with a chemotherapeutic class 1 HDAC inhibitor, romidepsin (FK228), at a low dose [(0.56 mg/kg twice a week) for 8 weeks], decreased blood glucose reduction independent of plasma insulin level. These authors have suggested that these anti-diabetic effects of romidepsin were mediated through HDAC2-mediated potentiation of intracellular insulin signaling (59).

Thus, available information till date suggests that HDAC inhibition has beneficial effects in ameliorating diabetic microvascular complications by targeting multiple dysregulated pathways. However, its translation into an effective therapy requires further studies such as evaluating association between HDACs and environmental and genetic factors.

## DNA Methylation in Diabetes-Induced Microvascular Complications

DNA methylation involves methylation at 5′ position of cytosine residues in CpG islands, mostly in the promoter regions and is carried out by DNA methyl transferases (DNMTs). Promoter DNA methylation is an important epigenetic mechanism regulating gene expression and is known to be affected in various diseases, including cardiovascular diseases and diabetes (60). Altered DNA methylation of inflammatory genes, glucose, and lipid metabolism genes, genes involved in oxidative stress, has been reported in diabetes (61).

DNA methylation in vascular complications of diabetes have been investigated and reviewed in a recent review (62). El-Osta (63) reported that short-term exposure of aortic endothelial cells to high glucose-induced promoter DNA methylation of *NF-kB p65* subunit, an important mediator of cardiac fibrosis. These authors showed that DNA methylation was mediated by hyperglycemia-induced increased methylglyoxal generation. Pirola et al. (35) observed that hyperglycemia significantly affects human vascular chromatin resulting in differential methylation and acetylation pattern with the transcriptional upregulation of genes involved in metabolic and cardiovascular disease. A good correlation was seen between hyper-acetylation and DNA methylation and induction of genes in glucose-treated cells, and suggested that hyperglycemia-induced gene induction was mediated by distinct changes in methylation and acetylation pattern of the genes.

Distinct promoter methylation profiling has been reported in diabetic hearts too. Movassagh et al. (64) examined DNA methylation profiles in left ventricular tissues from patients with idiopathic and end stage heart failure and observed increased promoter methylation of 3 genes, *PECAM1, ARHGAP24*, and *AMOTL2*, related to angiogenesis in cardiomyopathic hearts, suggesting a role of DNA methylation-induced altered gene expression in cardiomyopathy.

However, DNA methylation pattern seen in diabetic hearts is distinct from that seen in heart failure patients (9). A specific DNA methylation CpG site of β-myosin heavy chain (β*-MYH7*) gene that was found to be extensively methylated in T2DM hearts as compared to controls and 3 CpG sites of failing human hearts. Similar DNA methylation changes were also seen T1DM hearts and in steroid induced diabetic hearts (9), suggesting altered DNA methylation of specific CpG site of β*-MHC* may be contributing to ventricular dysfunction seen in diabetic patients.

In a similar study, Mönkemann et al. (65) reported altered methylation status of P53-inducible *p21WAF1/CIP1* promoter, resulting in activation of apoptotic pathway leading to cell death of cardiomyocytes and cardiomyopathy in diabetic rats. They proposed that oxidative stress was the major trigger contributing to *de novo* methylation of p53-inducible *p21WAF1/CIP1* gene.

Diabetes-induced oxidative stress is an important mediator of diabetes-associated cardiovascular complications. Zhong et al. (66) recently reported significant hypomethylation of *KEAP1* promoter in diabetic cardiomyopathy patients, with concomitant increase in KEAP1 protein levels in these patients. KEAP1 protein is known to bind to NF-E2-related factor 2 (NRF2), and promotes its degradation. NRF2 is known to activate several antioxidant enzymes. Studies have proposed that reduction of NRF2 antioxidant system in diabetic hearts may alter redox balance and contribute to increased oxidative stress in the heart of diabetic patients (67).

Vecellio et al. (68) have recently reported a decreased proliferation, differentiation potential, and premature cell death of cardiac mesenchymal stem cells in T2DM patients. Furthermore, they observed hypermethylation of promoter CpG islands of genes of cell cycle and DNA repair genes along with reduced acetylation of histone H3 lysine 9 (H3K9Ac) and lysine 14 (H3K14Ac) and increased trimethylation of H3K9Ac and lysine 27. They proposed that reduced HAT activity in diabetic hearts was responsible for increased DNA CpG methylation resulting in decreased cell differentiation and proliferation of cardiac mesenchymal stem cells in diabetic cardiomyopathy. However, these effects could be reversed by increasing HAT activity, suggesting a potential therapeutic application of epigenetic modulators in diabetes-associated cardiovascular complications.

Decreased promoter methylation of liver X receptor α (*LXRa*) (69) and *AT1b* angiotensin (67) receptor gene leading to their increased expression has been observed in diabetic hearts. TNF-α-mediated increased promoter methylation of sarcoplasmic reticulum Ca-ATPases (*SERCA2a*) resulting in decreased SERCA2a expression has been observed in high glucose-treated cardiomyocytes (70). These results suggest that diabetic milieu can cause increased or decreased methylation of different genes, resulting in their aberrant expression in heart.

Altered methylation of several genes dysregulated in diabetic nephropathy and diabetic retinopathy has been reported in diabetic patients and *in vitro* studies (35, 71, 72). A distinct differential promoter DNA methylation pattern has been reported in diabetic nephropathy patients with end-stage renal disease as compared to those who do not progress to this stage (73), suggesting that diabetic environment results in distinct epigenetic changes in specific genes, which could be used as prognostic biomarkers. Similarly, in diabetic retinopathy, Agardh et al. (74) reported differential DNA methylation of nearly 233 unique genes, with genes from natural killer cell-mediated cytotoxicity pathway genes to be hypomethylated in proliferative diabetic retinopathy (PDR) and suggested that this distinct methylation pattern could be used as a prospective marker of PDR. Mishra and Kowluru (75) have shown that increased DNA methylation of mitochondrial DNA (mtDNA) causes decreased transcription of mtDNA, impairing mitochondrial functions and increasing apoptosis in diabetic retinopathy. A dynamic balance between methyl cytosine and hydroxyl methylation of MMP9 was found to be important in MMP9 expression and in maintaining mitochondrial integrity and function in RECs and in preventing diabetic retinopathy (76). Diabetes-induced oxidative stress appears to be a major trigger of these epigenetic changes.

Thus, there is substantial evidence to suggest that hyperglycemia causes aberrant methylation of regulatory regions of several distinct genes resulting in their dysregulated expression. These molecular changes appear to be important in the pathogenesis of diabetes-induced microvascular changes in heart, kidney, and retina of the diabetic patients. Furthermore, diet, exercise, environment, and genetic factors, which are important contributors to risk of diabetes, are also potent modulators of epigenetic changes. Hence, their role in inducing epigenetic changes in microvascular complications in diabetic milieu needs to be explored.

## Non-Coding RNAs and Diabetes

Non-coding RNAs are non-protein coding RNAs, and include microRNAs, long non-coding RNAs (LncRNAs), circular RNAs, etc. have been identified as important regulators of gene expression. These molecules have been shown to be important in developmental, physiological, and pathological processes. Dysregulated expression of microRNAs and long non-coding RNAs (lncRNAs) has been implicated in various diseases, including vascular complications of diabetes.

## MicroRNAs Associated with Diabetes-Induced Cardiomyopathy

MicroRNAs are small non-coding RNAs which regulate gene expression by mRNA degradation or translational repression of mRNAs. The role of microRNAs has been widely studied in diabetes and its vascular complications and has been reviewed recently in several articles (17, 77, 78). Dysregulated expression of several microRNAs has been reported in Diabetic cardiomyopathy, retinopathy, nephropathy, and neuropathy regulating genes involved in diabetes (Table 1). Most of these microRNAs are involved in fibrogenesis, hypertrophy, apoptosis, inflammation, angiogenesis, and ECM accumulation. These functions are mainly regulated by microRNAs by regulating the expression of target genes involved in these cellular processes.

**Table 1:** Dysregulated microRNAs in microvascular complications of diabetes.

MicroRNAs	Targets	Functions	Reference
**Nephropathy**			
miR-192	TGF-β	ECM	(53)
miR-200b/c		Collagen, fibrosis	(46)
miR-21	PTEN	Renal cell hypertrophy and Fibrosis	(8)
miR-195	Bcl-2	Podocyte apoptosis	(70)
miR-377	Fibronectin	Fibrosis	(79)
miR-29 family	Collagen I, III, IV	Fibrosis	(53)
miR-93	VEGF-A	Glomerular function	(80)
**Retinopathy**			
miR-146, miR-155, miR-132, miR-21 (upregulated in retina)	Nf-κβ	Pro-apoptosis of retinal pericytes	(81)
miR-17-5p, miR-18a, miR-20a, miR-21, miR-31, miR-155 (upregulated in retinal endothelial cells)	Vascular endothelial growth factor	Vascular permeability	(82)
miR-200b	VEGF-A	Vascular permeability	(83)

**Figure 2:**
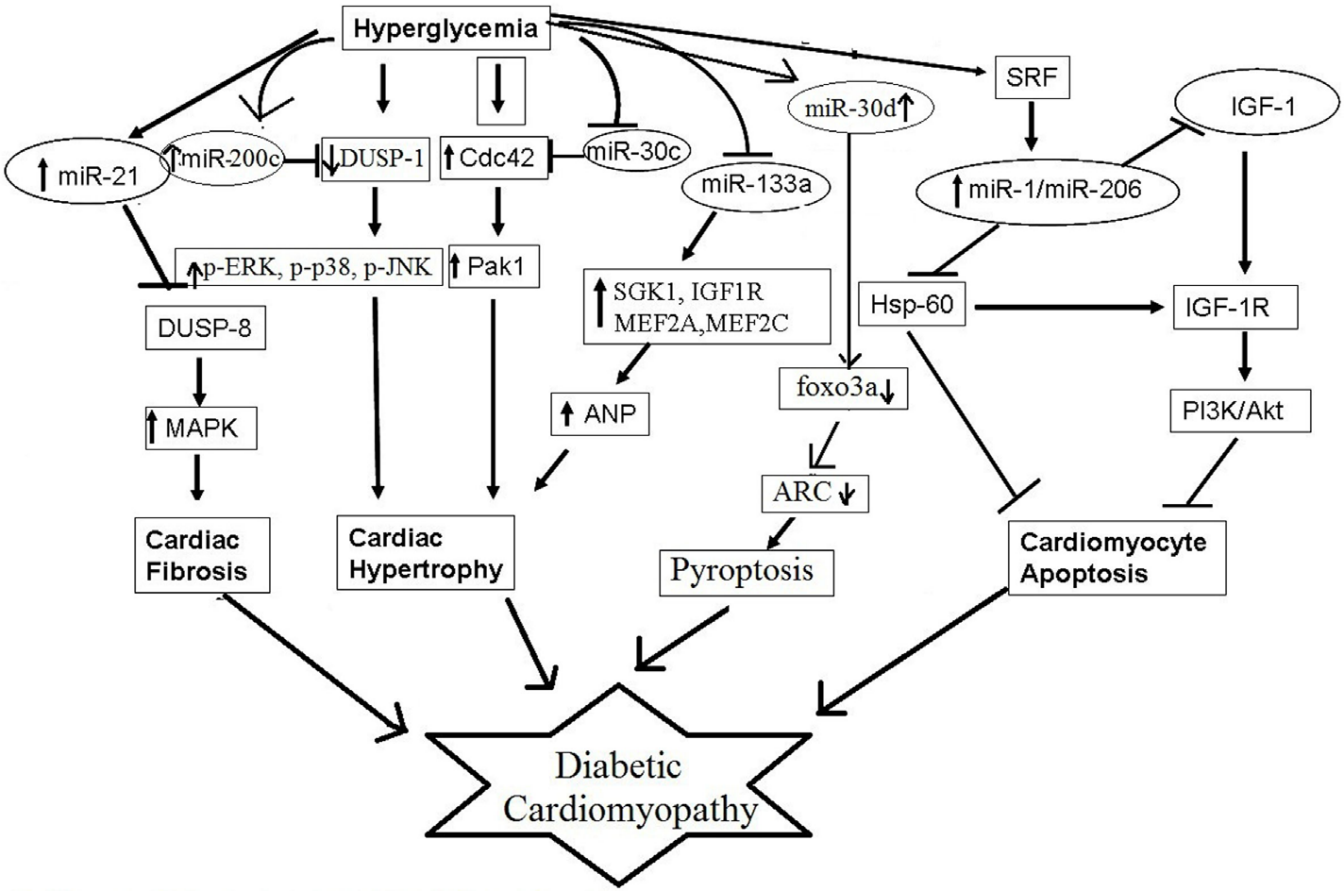
Schematic model of epigenetic role of microRNAs in diabetic cardiomyopathy.

Several microRNAs have been reported to contribute to pathophysiological processes of diabetic cardiomyopathy, such as myocardial fibrosis, cardiomyocyte hypertrophy, cardiomyocyte apoptosis, and mitochondrial dysfunction (Figure 2). For example, miR-30c, miR-133a, miR-150, and miR-373 were found to be downregulated and miR-451 was found to be upregulated in diabetes-induced cardiomyocyte hypertrophy (84). Whereas, in diabetes-induced cardiac fibrosis, the expression of miR-133a was found to be decreased and the expression of miR-21 was significantly increased (84). miR-34a, miR-1, miR-206, miR-195, and miR-30d have been implicated in diabetes-associated cardiac apoptosis and mitochondrial dysfunction (84). Raut et al. showed that the expression of putative target genes of miR-30c (*CDC42* and *PAK1*) were increased in hearts of diabetic rats and in HG treated cardiomyocytes (85, 86). miR-30c overexpression attenuated hyperglycemia-induced cardiomyocyte hypertrophy, whereas miR-30c inhibition resulted in myocyte hypertrophy in high glucose-treated cardiomyocytes, suggesting anti-hypertrophic potential of miR-30c in diabetic cardiomyopathy (85). miR-200c has been found to be pro-hypertrophic and its expression was shown to be significantly increased in diabetic hearts and in high glucose-treated cardiomyocytes. It was found to induce diabetes-associated cardiac hypertrophy by down regulating expression of dual-specific phosphatase-1 (DUSP-1). Inhibition of miR-200c augmented the expression of the DUSP-1 causing decreased expression of phosphorylated ERK, p38, and JNK and attenuated cardiomyocyte hypertrophy in high glucose-treated neonatal rat cardiomyocytes (87). In another study, miR-133a expression was reduced in diabetic cardiomyopathy along with augmented gene expression of *MEF2A, MEF2C, SGK1*, and *IGF1R*. Over expression of this microRNA inhibited altered gene expression and hypertrophic changes, indicating that miR-133a participated in mediating glucose-induced cardiomyocyte hypertrophy in diabetes (88). Duan et al. (89) reported significantly reduced expression of miR-150 in high glucose-treated cardiomyocytes; this microRNA was shown to increase p300 expression, resulting in cardiomyocyte hypertrophy. miR-373 has also been shown to be involved in the pathogenesis of diabetes-induced cardiac hypertrophy. The expression of miR-373 was found to be markedly down regulated in STZ-induced diabetic mice, and neonatal rat cardiomyocytes in response to high glucose. Over expression of miR-373 in cardiomyocytes using synthetic miR-373 mimics resulted in decreased expression of *MEF2C* gene and attenuated cardiomyocyte hypertrophy in high glucose-treated cardiomyocytes (90). Kuwabara et al. identified calcium-binding protein 39 (CAB39), a component of AMPK signaling pathway as direct target of miR-451. They demonstrated that in miR-451 knockout mouse the protein expression of CAB39 and phosphorylated AMPK was increased significantly, indicating that miR-451 was involved in diabetic cardiomyopathy *via* suppression of the LKB1/AMPK signaling pathway (91).

Several miRNAs, such as miR-21 and miR-29, have been shown to promote cardiac fibrosis in diabetic hearts. Liu et al. demonstrated increased miR-21 expression after high glucose treatment in cardiac fibroblasts (92). miR-21 was shown to promote fibroblast survival by down regulating SPRY1 (93). Silencing of miR-21 using synthetic mimics in mouse model of diabetic cardiomyopathy resulted in decreased interstitial fibrosis, suggesting potential role of miR-21 in cardiac fibrosis associated with diabetic cardiomyopathy. Liu et al. also showed that gain and loss of miR-21 function negatively regulated expression of DUSP8, a MAPK phosphatase, and enhanced cell proliferation and collagen synthesis *via* MAPK signaling pathway (92). Kumar et al. (94) have reported that miR-21 may also promote cardiac fibrosis by activation of AKT/PKB signaling. In addition to cardiac hypertrophy, miR-133a was found to mediate diabetes-induced cardiac fibrosis. Chen et al. observed that miR-133a expression was significantly decreased in hearts of STZ-induced diabetic mice, along with increased expression of transcriptional co-activator p300, as well as fibrosis markers (95).

miR-1 and miR-206 are cardiac-specific microRNAs (96). Increased miR-1 and miR-206 levels have been observed in high glucose-treated cardiomyocytes. Both these miRNAs were proposed to induce cardiomyopathy by inducing mitochondrial dysfunction and apoptosis (97). These microRNAs have been shown to bind to the same site in the 3′-UTR of *HSP60* mRNA and thereby could regulate HSP60 expression and glucose-mediated apoptosis in diabetic myocardium; however, this needs experimental validation (97). miR-34 too has been shown to promote HG-induced apoptotic changes in H9C2 cells (96). Pyroptosis is pro-inflammatory programmed cell death and is unlike from apoptosis or necrosis (26). Li et al. in their study showed that miR-30d expression was substantially increased in diabetic cardiomyopathy and this increased expression promoted cardiomyocyte pyroptosis; conversely, knockdown of miR-30d attenuated it (98).

It has been proposed that diabetic milieu may induce or repress the microRNAs by several different mechanisms, such as oxidative stress and ER stress, epigenetically regulating genes coding for microRNAs.

Role of microRNAs in diabetic nephropathy has been investigated widely and there are several recent reviews on this topic (99). Existing literature supports a pathogenic role for several microRNAs by promoting renal fibrosis by increased accumulation of extracellular matrix proteins related to fibrosis, glomerular hypertrophy, and renal cell apoptosis (100). Some of these microRNAs have been shown to have a potential as biomarkers as these were found to be dysregulated in early stages of nephropathy. However, more evidence is required for these data to be translated to clinical application. Furthermore, it has been observed that modulation of these microRNAs with either mimics or antagomiRs could attenuate the disease, suggesting that these microRNAs could be potential therapeutic targets.

A differential microRNA expression has been reported in patients with diabetic retinopathy as compared to controls. Animal and *in vitro* studies on RECs too showed altered retinal microRNA profile in diabetic animals and RECs treated with high glucose (35). Zampetaki et al. (101) showed that miR-27b and miR-320a increased risk of diabetic retinopathy by repressing antiangiogenic thrombospondin-1. Qin et al. have reported decreased miR-20b and correlated increase in its target genes VEGF and AKT3 in the retina and RECs in diabetic rats. These authors suggested that hyperglycemia-induced changes in retinal tissues were mediated by miR-20b *via* modulating VEGF and AKT3 in diabetic retinopathy (102). Similarly miR-15a too has been found to be protective toward developing diabetic retinopathy by inhibiting pro-inflammatory and pro-angiogenic pathways through its target genes ASM and VEGF-A (103). In a recent study, Zhou et al. (104) showed that transgenic mice over expressing let-7 show features similar to non-proliferative diabetic retinopathy suggesting its pathological role in non-proliferative diabetic retinopathy.

In addition, genetic variants in microRNA genes, such as miR-4513 rs2168518, miR-499 rs3746444, miR-196a2 rs11614913, and miR-423 rs6505162, have also been shown to be associated with the risk of cardiovascular complication of diabetes (105).

Taken together, available literature shows a definitive role of microRNAs, specifically targeting pro-angiogenic and pro-inflammatory genes in pathogenesis of diabetic retinopathy, thus providing their therapeutic potential in preventing and treatment of diabetic retinopathy.

## MicroRNAs as Biomarkers of Diabetic Vascular Complications

As serum levels of different miRNAs have been shown to be elevated in cardiovascular complication of diabetes, they could serve as sensitive and cost-effective biomarkers for these conditions. For example, the expression levels of seven diabetes-related miRNAs (miR-9, miR-29a, miR-30d, miR-34a, miR-124a, miR-146a, and miR-375) in serum were shown to be significantly elevated in T2DM subjects compared with pre-diabetes and/or normal glucose tolerance suggesting that during the pathogenesis of T2DM, the peripheral diabetes-related miRNAs have not changed significantly from susceptible individual with normal glucose tolerance at pre-diabetic stage (106). miR-1 and miR-133a have been found to be good predictors of myocardial steatosis in diabetic patients (105). The fact that miR-1 and miR-133a are poorly associated with other clinical, biochemical, metabolic, hemodynamic, and cardiac parameters, and even with verified absence of clinically evident myocardial ischemia and/or damage supports the hypothesis that these miRNAs are independent predictors of myocardial steatosis.

miR-21, miR-29a/b/c, and miR-192 could reflect DN pathogenesis and serve as biomarkers during DN progression as there levels were significantly enriched in the overt proteinuria group compared with microalbuminuria and/or overt proteinuria groups. Authors observed that miR-192 suppressed the translation of SIP1/E-box repressors ZEB2, leading to elevated collagen deposition *in vivo* indicating a role of miR-192 in the development of the matrix accumulation observed in DN. Whereas, miR-21 prevented mesangial hypertrophy by targeting the PTEN/PI3K/AKT pathway and miR-29 was negatively regulated by TGF-β1 *via* SMAD3 signaling pathway, thereby promoting collagen matrix expression (107).

Some microRNAs have been found significantly increased in blood samples of diabetic patients with retinopathy; for example, Qing et al. (108) have reported that circulating miR-21, miR-181c, and miR-1179 together could be good biomarkers for differentiating between proliferative and non-proliferative retinopathy. Barutta et al. (109) recently reported that circulating miR-126 levels were significantly lower in diabetic patients as compared to controls and were associated with both micro- and macrovascular complications, especially with proliferative retinopathy In a large cohort of type 1 diabetic subjects. Circulating microRNAs as biomarkers of diabetes-induced cardiomyopathy have also been reviewed recently (110).

Exosomes are small extracellular vesicles present in blood and urine is rich in microRNAs and is being investigated as potential disease markers. Mohan et al. (111) showed that urinary exosomal microRNAs 451-5p levels increased and correlated with renal damage in diabetic rats and suggested these to be useful as early biomarkers of diabetic nephropathy. Thus, microRNAs show promising potential as biomarkers for vascular complications of diabetes.

## MicroRNAs as Therapeutics in Microvascular Complications of Diabetes

MicroRNAs have been explored for their potential as new therapeutic targets in diabetes vascular complications; for example, Kovacs et al. (82) showed that miR-146 through its inhibition of NF-kβ activation could be a potential therapeutic target in cardiovascular complication of diabetes. miR-130a is shown to improve EPCs function by negatively regulating RUNX3 and through ERK/VEGF and AKT pathways and could have a potential use in improving endothelial function (112). Downregulation of miR-200b has been implicated in Glucose-induced augmented vascular endothelial growth factor (VEGF) production through histone H3 lysine-27 trimethylation (113). Thus, methyltransferase inhibitors like COMT inhibitor could be used to control VEGF augmentation by upregulation of miR-200b. On similar grounds, in a recent study, vitamin B_3_ and nicotinic acid have been shown to have a protective effect in diabetic retinopathy by upregulating miR-126 (114). miR-34 family modulates changes in proliferation and migration of retinal pigment epithelial cells through downregulation of leucine-rich repeat-containing G-protein coupled receptor 4 (LGR4) expressions, indicating G protein (heterotrimeric) inhibitors as potential therapeutics (115). Apart from this, miR-21 is an important miRNA frequently upregulated in T2DM and cardiovascular complication of diabetes (116). miR-21 targets SMAD7 pathway and also blocks the expression of PDCD4 and thereby, suppress activation of the TGF-β and NF-κB signaling pathways. Since miR-21 is upregulated in cells related to diabetic complications, their exclusive molecular signatures can be used as prognosis, diagnosis, and therapeutic targets. Sekar et al. (79) have also shown that targeting miR-21 by synthetic anti-miRNA oligonucleotides (AMOs) with 2-O-methylmodification effectively inhibited the miRNA 21 in cell culture and xenograft mouse models. In addition to this, antisense-RNA, miRNAs mimics, and tumor suppressor miRNAs could be also used to inhibit the expression of miR-21.

## Long Non-Coding RNAs and Vascular Complications of Diabetes

Long non-coding RNAs (lncRNAs) are >200-nt-long non-coding RNAs and are increasingly being recognized as important gene regulators. lncRNAs repress gene expression by binding to specific DNA/RNA or protein moieties (80). For example, they can bind to miRNAs and thereby prevent their binding to target mRNAs and, hence, gene expression (81) or they may regulate activity of regulatory proteins by altering their affinity or cellular localization for other proteins (83). Aberrant expression of lncRNAs has been implicated in pathophysiology of several diseases such as tumorigenesis and cardiovascular diseases; however, their role in vascular complications of diabetes remains largely unknown. Recent studies have identified several lncRNAs with potential role in diabetic nephropathy, retinopathy, neuropathy, and cardiomyopathy.

Data on lncRNAs in diabetic cardiomyopathy are sparse. Zhang et al. (117) recently reported increased expression of lncRNAs MALAT1 in the heart tissue of diabetic rats, and observed that its inhibition Improved left ventricular function, by attenuating cardiomyocyte apoptosis. A downregulation of lncRNA H19 has been also seen in diabetic hearts and it has been shown to increase expression of miRNA-675 and downregulation of its target VDAC1 leading to decreased cardiomyocyte apoptosis of cardiomyocytes in high glucose milieu (118). Zhuo et al. (119) have recently showed that H19 also inhibited autophagy in glucose-treated cardiomyocytes by silencing pro-autophagy DIRAS3. lnc H19 has been suggested as a potential biomarker and therapeutic target for diabetic cardiomyopathy. However, more research is needed to explore the potential role of lncRNAs in diabetic cardiomyopathy.

Wang et al. (120) reported downregulation of CYP4B1-PS1-001 in both in early stages of diabetic nephropathy and suggested its role in mesangial cell proliferation and fibrosis. Alvarez et al. (100) earlier showed that a long non-coding RNA, the plasmacytoma variant translocation 1 (PVT1), increased fibronectin 1 (FN1) ECM accumulation in the glomeruli under hyperglycemic conditions, suggesting its role in diabetic nephropathy. They recently reported that miR-1207-5p, a PVT1-derived microRNA, was also independently involved in pathogenesis of diabetic nephropathy. Similarly lncRNA ENSMUST00000147869 associated with Cyp4a12a has been shown to mediate diabetic nephropathy by increasing proliferation and fibrosis of mesangial cells (121). Several other lncRNAs, such as MALAT1 (122), myocardial infarction-associated transcript (MIAT) (123) and lnc-MGC, have been found to be dysregulated in diabetes-induced renal injury and are potential therapeutic targets for treating diabetes-induced nephropathy.

lncRNA-RNCR3 has been implicated in diabetes-induced retinopathy. Liu et al. (67) recently showed that lncRNA-RNCR3 knockdown decreased cytokine levels, retinal cell apoptosis, improved visual function, and inhibited retinal reactive gliosis in diabetic animals, indicating its role in diabetes-induced neurodegeneration. Shan et al. have increase in RNCR3 levels following high glucose stress both *in vitro* and *in vivo*. They observed that RNCR3 knockdown inhibited RECs proliferation, and cell migration and tube formation *in vitro* and improved endothelial function *in vivo* (124) *via* RNCR3/KLF2/miR-185-5p pathway, suggesting RNCR3 inhibition as a therapeutic option in treating diabetic retinal abnormalities.

The studies done so far indicate that lncRNAs are important mediators of various vascular complications of diabetes and potential therapeutic targets and need to be explored further.

## Concluding Remarks

Metabolic disorders such as diabetes are due to cumulative interactive effects of genetic and environmental factors. These effects are primarily induced by diabetes-associated factors, such as hyperglycemia, oxidative stress, inflammation, obesity, and so on, and are manifested as epigenetic changes in the genome. These epigenetic changes include DNA methylation, histone methylation and acetylation, deregulated expression of microRNAs and lncRNAs etc. and are responsible for altered gene expression of the key regulatory pathways mediating diabetes-associated vascular complications and also are major contributors to metabolic memory associated with diabetes. Thus, study of epigenetic mechanisms assumes a significant role in elucidating pathophysiology of diabetes and its complications. However, our understanding of these mechanisms is incomplete and awaits translational application. Further research focus is needed to elucidate the mechanisms especially with respect to non-coding RNAs and chromatin structure. The information being generated in microRNAs and lncRNAs shows that we are at threshold of unveiling of important biological role of these molecules in disease etiology, pathology, progression, and therapeutics, besides being non-invasive diagnostic and prognostic biomarkers of vascular complications, such as nephropathy and cardiomyopathy.

To gain a deeper understanding of T2DM and its associated microvascular complications, an incorporation of a range of novel tools and techniques, such as RNAseq, transcriptomics, metabolomics, epigenomic profiling, and chromatin 3D mapping, is needed to be integrated in diabetes research. Tissue- and cell-specific profiling of methylation levels and histone modifications of major pathophysiological genes would increase our understanding of the pathology of T2DM and associated complications. Elucidation of association between epigenetic modulations of the genome involved in microvascular complication with those of macrovascular complications of diabetes is also needed. The knowledge gained through epigenetics gene expression alteration in diabetic cardiomyopathy will provide better approaches in attenuating hyperglycemia-induced damage to the heart and other affected organs, such as kidney and brain. Thus, elucidation of epigenetic mechanisms in conjunction with environmental and genetic factors would fine tune the understanding of pathophysiology of diabetic cardiomyopathy. And, epigenetic factors could provide a wholesome picture of the role of genes and their expression in T2DM and its micro as well as macrovascular complications.

## Author Contributions

MK: checking, editing, re-writing, data approval, and guarantor of work. BC: data collection, writing, and reference updation. SR: checking, editing, data collection, writing, and reference updation.

## Conflict of Interest Statement

The authors declare that the research was conducted in the absence of any commercial or financial relationships that could be construed as a potential conflict of interest.
